# Boston Letter

**Published:** 1879-11

**Authors:** 


### Domestic Correspondence.

### ARTICLE XIII.

### BOSTON LETTER.

MESSRS. EDITORS:—If we include dispensaries with hospitals, Montesquieu's remark, "Malheur au pays qui a beaucoup d'hôpitaux," will apply as strictly to Boston as to any other American city. For charitable medicine is in strong force here. Good coats and bonnets, with feathers in them, encouraged by the liberality with which advice and medicines are dispensed, ask for gratuitous treatment here as frequently as do similar frauds in other cities. But do not for a moment imagine that the growth of this evil has escaped watchful eyes. Benjamin Franklin once said: "In my youth I traveled much, and observed, in different countries, that the more public provisions were made for the poor, the less they provided for themselves." The principle which underlies this observation has become so strongly ingrained in the minds of those who control the charities of Boston, that an unceasing effort is made to cultivate more pride and a feeling of greater independence in the minds of those who apply for aid. In our dispensaries watchfulness is exercised lest gratuitous advice and medicines be given to individuals who are able to pay for them. To suspected patients, opportunity is given to pay a fee, no matter how small in amount. The practice is a wise one, and in the process of time must effect a healthful influence. But it is amazing, particularly when money is scarce, to see how easily individuals quite above the ordinary dispensary patient, can pocket their pride—if indeed they possess such a commodity—and calmly apply for aid for which they are perfectly well able to pay a moderate fee. Unless they be met with a wise firmness, it is hardly necessary to say that not only does their manhood or womanhood become seriously injured, but the city becomes unfairly and increasingly burdened. With some exceptions, which, however constant, should be but few, charity of every sort ought to be so dispensed that while the objects of it receive aid, they at the same time should not be allowed to feel that they can freely secure something for nothing. Indeed, in the old Boston dispensary, to a brief history of which the foregoing remarks are preliminary, an official has been appointed whose business it is to separate the sheep from the goats.

"In order that the funds needed for the worthy poor may not be applied to relieve persons who have sufficient means to purchase their own medicines," says the dispensary report for 1878–79, "it has been found necessary to discriminate carefully between the patients asking for assistance. The managers have accordingly chosen an officer who assists the superintendent by inquiring into the condition of the applicants.

"After careful investigation, he notified certain persons that they were able to pay for their own medical attendance, and others that this was a Boston institution, not chartered for the residents of other towns in this or adjoining States."

This brave old dispensary, the only one we have which is not an out-patient department of some one of the hospitals, ranks number four in the list of all existing charities of a similar nature, the first having been established in London in 1770, under the auspices of Dr. Hulme; the second in Philadelphia in 1786 (and which, by-the-bye, is run to-day just as it was nearly one hundred years ago, much to the loss of students of medicine); the third in New York in 1786, about which I know nothing; the fourth, our own, in 1796.

For many years patients were obliged to bring a contributor's letter couched in the following terms:

"*To the Physicians of the Boston Dispensary:* I recommend ———————— to the care of the Dispensary, believing —— to be a proper object of this charity. ———, *Contributor.*"

And the patient, after recovery, received a ticket of discharge.

These formalities were discontinued in the year 1856, since which time patients have applied and been treated without introduction.

In 1852 the dispensary was empowered to supply food, fuel, clothing, "or any other kind of relief," in addition to medical assistance, to suffering applicants. In this respect the institution, however, is peculiar, and its charities, therefore, are much broader than those of the ordinary and purely medical dispensary. The house occupied by this charity is a plain, two story and square brick building, of Spartan simplicity within and without. It stands in a quiet by-street, and in view of the amount of relief given within its walls, would seem too contracted.

In some cities, notably Philadelphia, dispensaries have a resident physician who is supposed to be such an Admirable Crichton of medical science that he, single-handed, can cope with every ill which may present itself among the thousands of yearly applicants for relief. In the management of the Boston Dispensary there is more modesty if not less wisdom. As the patients apply, their ails are sifted, so that the child goes to one department, a skin case to another, an eye disease to a third, surgical cases to a fourth, women's diseases to a fifth, and so on. That is, the treatment is split up into specialties, as it ought to be, and at the head of each department is a competent and well-tried physician or surgeon. For, in Boston, it is considered an *honor* to be a dispensary medical official. And not only are the attending physicians and surgeons men of experience and men of name, but the district physicians are young men who rank high in their classes, and who are as anxious to secure the position as they are in later years to obtain State appointments in hospitals. So that with us dispensary work, instead of being looked down upon, is sought by the very best men. This is as it should be, and, as a consequence, it need not be said that our dispensary is admirably conducted. The patients receive treatment from a staff consisting of four surgeons and nineteen physicians, of whom the requisite number are in daily attendance in their several departments. There are likewise nine district physicians, whose work is that of all outside physicians among the poor. But while in some American cities the district physician receives no other remuneration than such experience as he may gain — (this, by the way, has always struck me as being a very short-sighted and unjust arrangement) — in Boston he receives $200 per year during his term of service. The dispensary is in charge of a superintendent, an apothecary and his assistant, all of whom receive fair salaries, the whole being supervised as is usual by a board of managers.

In the report of 1878 it may be seen that 22,694 new and about 30,000 old patients were treated at the dispensary, and that there were 17,160 district patients, among whom the death-rate was but 3.2 per cent., which is a most excellent showing. The entire cost of treating these sixty-nine thousand and odd hundred patients was only $8,977.46.

One of the regulations of the dispensary is the following:

"ART. 5. *Medical students shall have the privilege of attending at the office of the dispensary*, but shall in no case be permitted to prescribe for or operate on any patient. The privilege thus granted shall be for the space of one year, and may be interdicted at any time by the executive committee."

Of itself this regulation renders the dispensary a most valuable institution, for, in a short space of time a student is made familiar with a wide variety of cases. He not only sees and can examine each case, but the treatment as well. And knowing the diagnosis, he can easily acquaint himself with the leading symptoms. Moreover, the physicians of the dispensary are at liberty to form private classes of students, and, having large numbers of patients from which to choose, they can fairly illustrate every subject of the course. This is especially true of the courses in auscultation and percussion. So that students of the Harvard school may make themselves practically familiar with the proper manner of working out a diagnosis. They not only have a clear presentation of the theories of a given case, but can also study and memorize symptoms upon the patient.

This is a wise and beneficial use of the dispensary patients, which in the dispensaries of other cities is not permitted. Consequently, students not being admitted, a vast quantity of clinical material which should be but is not utilized for the instruction of medical students, goes to waste.

As an illustration of this mistaken policy, I may mention an instance in which application was made to the old Philadelphia Dispensary, for permission to use the patients in a private course in chest diseases. Permission was refused, because of the antiquated and foolishly conservative policy of the board of managers. Indeed, but for a sort of shrewd game of euchre, Strawbridge would never have managed to get possession of the many eye cases which have created the excellent success of his department of this dispensary. As it was he very nearly failed.

We have a so-called "Consumptives' Home" in Boston, which is very curiously managed by its head, Dr. Charles Cullis. It includes a "spinal home" for spinal patients; a "cancer home;" a "faith cure home" for the temporary care of patients coming from a distance; children's homes, which care for children of patients until the parents recover. If they die, the children are adopted by the Home. In the advertisement of this institution its therapeutics and means of support are announced in the words: "*Cures by Prayer. Supported by Prayer.*" The manager, Dr. Cullis, it is said, seeks no financial aid except only and wholly by prayer. It is affirmed that he never in any case has asked a single individual for money. However, this may be, the institution has grown from nothing to an extensive establishment. As to the efficacy of its therapeutics, I have just heard of a case in which a large abscess developed in the cervical glands of a consumptive inmate. Dr. Cullis refused to put in the knife, preferring to confine himself to prayer. Somehow, the poor sufferer was carried to the City hospital, where the prayer was answered through the hands of those who help themselves and thus receive help. After which the girl was returned to Dr. Cullis. All of which will be considered peculiar.

In a former letter I promised to advise you of the forthcoming discussion, in the councilors' meeting of the Massachusetts Medical Society, of the question of the admission of female practitioners to the examination for entrance into the society. The meeting and the discussion came off the other day. The former was well attended, and the latter was warm and breezy. Opinions were very diverse. But it happened that prominent members who formerly opposed the innovation, voted in favor of it. Others stood by their guns and fought hard against it. Still, it was thought best to discuss the matter fully and exhaustively, so that it might be settled once for all. It has hung fire a long time, and members were tired of it. The result was that the new departure received the vote of a majority, but a small one.

The question having been brought to an issue, it is thought that the effect of the decision will be practically trivial. In New York and Rhode Island, medical societies have opened their examinations to women, but less than a half dozen of the latter have been successful. In our State the Censors have made the examination so rigorous that it is by no means easy for a medical graduate to pass. I need not say that the stronger minds hope the noble old society will not lose its time-honored dignity. It will be severely tried by the admission of women, and it will not be surprising if many members refuse to attend the annual meetings. Notwithstanding the fact that the council is composed of one out of every eight members from all parts of the State, the very small majority is a strong indication that if the vote could be taken in general meeting, the result would be the reverse. One thing is absolutely certain—if the decision were left in the hands of the Boston members of the society, it would bring forth a decided NO.

On the other hand, if women are to be admitted, the question of their medical education at once comes to the surface. And this, perhaps, will re-open that other question viz: their admission to the Harvard Medical School on equal terms with men. Otherwise how can Massachusetts women secure a medical education of such quality as will fairly admit them to the society? In spite of the decision of the councilors of the society, this matter is by no means settled.

If the doors of the Harvard Medical School are ever opened to women, it will be by the overweening influence of non-medical committees, whose members do not begin to conceive what it means to unite young women with young men in the co-study of medicine, surgery and obstetrics.

No delicate, womanly woman can thus unsex herself. And the women who can associate themselves with men in a full course of medicine and surgery are precisely those who lose that chivalric respect which manly men feel for true womanhood.

Far be it from all broad minds to object to the adoption of a medical life by women, but let them have their own schools, otherwise female medical students will become a sort of germ, a distinctive class of women. * *

BOSTON, Oct. 15, 1879.

